# Comparative Analysis of the Mechanical and Biofilm Surface Formation of 3D Printed Resins and Milled PMMA Blocks With and Without Inorganic Fillers

**DOI:** 10.1002/cre2.70433

**Published:** 2026-08-13

**Authors:** Indyara Cerutti, Juliana S. R. de Andrade, Andressa da Silva Barboza, Jaqueline B. Machado, Dewey Du‐Hyeong Lee, Mateus Bertolini Fernandes dos Santos

**Affiliations:** ^1^ Graduate Program in Dentistry Federal University of Pelotas Pelotas RS Brazil; ^2^ Graduate Program in Dentistry Federal University of Santa Catarina Florianópolis SC Brazil; ^3^ Department of Prosthodontics, College of Dentistry and Dental Clinics University of Iowa Iowa City Iowa USA

**Keywords:** 3D printing, biofilm formation, CAD/CAM, flexural strength, PMMA

## Abstract

**Objectives:**

Despite the growing number of studies assessing the performance of temporary restorative materials fabricated through CAD/CAM technologies, comparative analyses among 3D printed resins and milled PMMA blocks with differing compositions remain limited. This study compared mechanical and biofilm surface formation of 3D printed resins PMMA blocks with different compositions, using composite resin as control.

**Material and Methods:**

Bar‑shaped (25 × 2 × 2 mm) and disc‑shaped (6 × 2 mm) specimens were fabricated. Flexural strength was evaluated by three‑point bending (ISO 4049), and Vickers microhardness was recorded. Multispecies biofilm formation was quantified using CFU/mL and examined morphologically via SEM. Statistical significance was set at *α* = 0.05.

**Results:**

The 3D printed resins and milled hybrid PMMA showed the lowest flexural strength, with no significant differences between them, while the composite resin achieved the highest and statistically superior values (*p* < 0.001). 3D printed resin without inorganic fillers showed the lowest Vickers microhardness values (12.66 ± 0.83 HK) while 3D printed resin with inorganic fillers (15.20 ± 1.94 HK) and milled conventional PMMA (16.03 ± 1.35 HK) exhibited higher values. However, none of them differed statistically from the 3D printed resin without inorganic fillers (*p* = 0.064). Conversely, milled hybrid PMMA (16.67 ± 2.10 HK) demonstrated significantly higher Vickers microhardness than 3 d printed resin with inorganic fillers (*p* < 0.001). Biofilm formation ranged from 2.26 × 10^7^ to 2.82 × 10^7^ CFU/mL, with no significant differences among materials (*p* > 0.05).

**Conclusions:**

Milled and 3D printed materials demonstrated comparable mechanical behavior while composite resin remained the performance benchmark. Material selection for provisional restorations should account for mechanical behavior and biological interactions, as surface treatment and clinical conditions may influence microbial colonization.

## 1 Introduction

Temporary crowns serve a critical function in prosthetic rehabilitation by maintaining occlusal stability, restoring masticatory function, preserving esthetics, and conditioning peri‑coronal soft tissues during the transition to the definitive prosthesis (de Souza et al. 2024). Regardless of their duration in the oral environment, temporary restorative materials must exhibit suitable mechanical and biological properties to ensure treatment predictability, minimize structural failures, and reduce the risk of inflammatory or peri‑implant complications associated with material degradation or microbial colonization (Alzahrani et al. 2023).

The integration of digital technologies into contemporary dental practice has facilitated the widespread adoption of computer aided/computed manufacturing (CAD/CAM) techniques for the fabrication of temporary restorations. These restorations may be produced either by milling industrially polymerized PMMA blocks (subtractive manufacturing) or by three‐dimensional (3D) printing light‐cured resins specifically formulated for temporary use (Sidhom et al. 2022). Each manufacturing method presents distinct advantages and limitations. Milled PMMA exhibits superior mechanical and dimensional stability but involves higher production costs and increased material waste (Al Wadei et al. 2022). In contrast, 3D printed resins have become increasingly popular due to improved equipment accessibility, reduced fabrication waste, and the capacity to generate complex geometries with efficiency (Alhallak et al. 2023). These differences underscore the need to investigate how material composition and manufacturing processes influence the mechanical and biological behavior of temporary restorations.

The performance of temporary materials is strongly influenced by the formulation of the organic matrix and the characteristics of incorporated inorganic fillers. Flexural strength is primarily governed by the chemical nature of the monomers and the degree of cross‑linking within the polymeric network, whereas surface hardness is determined largely by the concentration, morphology, and distribution of inorganic particles (Liang et al. 2025).

3D‑printing resins for provisional restorations can be broadly categorized as with or without inorganic fillers formulations, composed predominantly of light‐activated dimethacrylate monomers, and filler‐reinforced formulations incorporating inorganic fillers (e.g., silica or zirconia) to enhance mechanical performance (Liang et al. 2025). Milled materials are commonly supplied either as conventional PMMA blocks, consisting of high‑molecular‑weight acrylic matrices cross‑linked with agents such as ethylene glycol dimethacrylate (EGDMA), or as hybrid PMMA blocks containing inorganic fillers (e.g., silicon dioxide and aluminum oxide) to improve dimensional stability and wear resistance (Zhang et al. 2023). Beyond mechanical considerations, matrix chemistry and degree of conversion critically influence biocompatibility, residual monomer release, and bacterial adhesion, factors that bear directly on long‑term clinical performance and the risk of pathogenic biofilm development (Berghaus et al. 2023).

While previous studies have compared CAD/CAM and 3D‐printed provisional materials, most investigations have focused on either mechanical or biological outcomes in isolation, often without considering differences in material composition such as the presence of inorganic fillers. The present study is novel in that it simultaneously evaluates mechanical and biological behavior across 3D‐printed resins with distinct formulations, milled PMMA (conventional and hybrid), and a high‐performance composite resin under a unified experimental design. The inclusion of composite resin as a reference material was intended not as a conventional provisional benchmark, but as a high‐performance comparator, enabling contextualization of the mechanical limits achievable by polymer‐based restorative materials.

The null hypothesis was that temporary materials fabricated either by 3D printing or by milling PMMA blocks would not demonstrate inferior performance relative to the composite resin across all evaluated parameters.

## 2 Material and Methods

This in vitro study evaluated five restorative materials: two 3D printed resins (PriZma Bio Prov and Prizma Bio Crown), two PMMA blocks for milling (PMMA EVOLUX Blocks and PMMA EVOLUX Hybrid/Nanocomposite), and a conventional composite resin (Filtek Z350XT). The materials and their respective compositions are presented in Table 1.

**Table 1:** Materials included in the study, their manufacturer information, and detailed organic and inorganic composition as provided by the respective manufacturers.

Material	Manufacturer	Organic matrix	Inorganic filler	Detailed composition (manufacturer data)
3D printed resin without inorganic filler (Prizma Bio Prov)	Makertech Labs	Photopolymerizable monomers/acrylated and tri acrylated monomers	Amorphous silica	Acrylated and tri acrylated monomers, amorphous silica*, Diphenyl (2,4,6 trimethylbenzoyl) phosphine oxide, pigments
3D printed resin with inorganic filler (Prizma Bio Crown)	Makertech Labs	Methacrylic monomers/UDMA	Silica, zirconia, ceramic fillers	Methacrylated monomers, amorphous silica, UDMA, titanium dioxide, silanized zirconia, ceramic fillers, Diphenyl (2,4,6 trimethylbenzoyl)‐phosphine oxide
Milled conventional PMMA (Evolux PMMA)	Blue Dent	PMMA + EGDMA	Absent	PMMA, EGDMA, fluorescent pigments, organic pigments
Milled hybrid PMMA (Evolux Hybrid/Nanocomposite)	Blue Dent	PMMA + TEGDMA/Polymeric resin matrix 45%–60% (PMMA 40%–55%, dibenzoyl peroxide 0.5%–2%, TEGMA 3%–5%, carboxymethylcellulose 1.5%–2.5%)	Alumina, silicon dioxide, silanized glass nanoparticles	Silanized glass nanoparticles (40%–55%), alumina (5%–15%), polysiloxane (3%–7%), silicon dioxide (30%–45%)
Composite resin (Filtek Z350 XT)	3 M ESPE	Bis GMA, UDMA, TEGDMA, Bis EMA(6), PEGDMA	Silica and zirconia	Silanized ceramic fillers, Bis GMA, Bis EMA 6, UDMA, PEGDMA, treated silica, pigments

### 2.1 Sample Size and Specimen Preparation

Sample size was defined based on previous in vitro studies evaluating similar mechanical and biological outcomes and, when applicable, on statistical power considerations reported in the literature (Pantea et al. 2022; Lemus and Valm 2023). Consistent with established methodologies, *n* = 10 specimens per group were used for mechanical testing, while smaller sample sizes were adopted for microbiological and qualitative analyses (*n* = 6 for biofilm formation, *n* = 4 for CFU quantification, and *n* = 2 for SEM), as commonly reported in dental materials research.

All specimens were fabricated into two standardized geometries. Rectangular bars measuring 25 × 2 × 2 mm that were prepared for flexural strength testing, while disk‐shaped specimens with a diameter of 6 mm and a thickness of 2 mm were produced for the biofilm formation assays. The detailed fabrication procedures for each experimental group are described in the following sections.

### 2.2 3D Printed Resins

Digital models of bars and disks specimens were 3D printed using an LCD‑based Anycubic Photon Mono X 6Ks printer (Anycubic Inc.) with a 50‑µm layer thickness. Following printing, specimens were washed in 99% isopropyl alcohol for 5 min in an ultrasonic bath, air‑dried, and post‑cured in a 405‑nm UV chamber using the Anycubic Wash & Cure 2.0 system. Post‑curing times followed manufacturer protocols: 30 min for Bio Crown and 15 min for Bio Prov.

### 2.3 PMMA (Milled Groups)

Specimens were produced from pre‑polymerized PMMA blocks. Bars were sectioned using a diamond disk (EXTEC Dia) mounted on an IsoMet 1000 precision cutting machine (Buehler Inc.), under continuous water cooling. Cutting speed and pressure were standardized to minimize thermal fluctuations and residual stress. For disk fabrication, cylindrical segments were first obtained from the blocks and subsequently sectioned into 2‑mm‑thick disks using the cutting system under identical standardized conditions.

### 2.4 Composite Resin (Control)

Rectangular bar specimens were fabricated using incremental technique in a split stainless‑steel mold (Odeme Dental Research). A polyester strip and glass slide were placed over the material, applying slight pressure to create a uniform surface. Photopolymerization was performed at three different points with the light tip positioned 10 mm from the surface, using a VALO curing unit (Ultradent Products). For disks, a condensation silicone guide was fabricated from a 3D printed template. The stratification technique followed the same protocol used for the bar specimens.

### 2.5 Surface Finishing and Coating

Following fabrication, all specimens (bars and disks) were polished using sequential silicon‑carbide papers (400, 600, and 1200 grit) to obtain smooth and standardized surfaces. Dimensions were verified using a digital caliper (Mitutoyo Corp).

A layer of Palaseal glaze (Kulzer GmbH) was applied to CAD/CAM and 3D‐printed materials to simulate manufacturer‐recommended clinical finishing protocols. The composite resin group did not receive glaze, as this material is typically finished by polishing alone in clinical practice. However, this difference represents a potential confounding factor for biofilm analysis and should be considered when interpreting biological outcomes.

All specimens were finished using a standardized polishing protocol under controlled pressure and duration, following a sequential polishing with silicon carbide abrasive papers of decreasing grit size (400, 600, and 1200) (Standardization IOf 2019; Bollenl et al. 1997).

### 2.6 Flexural Strength Test

Flexural strength testing was performed in accordance with ISO 4049:2024. Ten rectangular bars from each group were immersed in distilled water at 37°C for 24 h prior to testing. A three‑point bending test was then carried out using a support span of 20 mm, a crosshead speed of 0.5 mm/min, and a maximum load of 1000 N. All tests were conducted using a universal testing machine (EMIC DL500). The flexural strength was obtained from the resulting stress–strain curve.

### 2.7 Vickers Microhardness Test

Microhardness was evaluated using fragments obtained from the same specimens used for the flexural strength test. Ten samples from each group were analyzed with a Vickers microhardness tester (Future‑Tech Corp). To avoid any influence of the three‐point bending test on the measurements, indentations were performed on regions that had not been in contact with either the loading tip or the supporting rollers during flexural testing. For each specimen, the initial microhardness value (D_0_) was determined by averaging three equidistant indentations spaced 150 µm apart. The measurements were performed using a 50 g load and a 5‑s dwell time.

### 2.8 Microcosm Biofilm Formation and Quantification

Six disks from each material (6 × 2 mm) were sterilized by UV irradiation for 30 min on each surface. Supragingival plaque was collected from a healthy adult volunteer (approved by local Ethics Committee, protocol HUM‑00164678) and suspended in Brain Heart Infusion (BHI) broth supplemented with 0.5% sucrose. After homogenization and 24‑hour aerobic incubation at 37°C, the bacterial concentration was adjusted to 7.5 × 10^7^ CFU/mL.

Disks (*n* = 6 per group) were placed in 24‑well plates containing 1.8 mL of BHI and 0.2 mL of the inoculum and incubated aerobically at 37°C for 7 days, with medium replacement every 48 h. After incubation, non‑adherent bacteria were removed with gentle rinsing in PBS. Specimens were then processed for colony‑forming unit (CFU) quantification and scanning electron microscopy (SEM).

For CFU counts (*n* = 4), each disk was transferred to a sterile tube containing 1 mL saline and vortexed for 60 s to detach the biofilm. Serial dilutions were plated on BHI agar and incubated aerobically at 37°C for 24 h. Colonies were counted and expressed as CFU/mL.

For SEM analysis (*n* = 2), specimens were fixed in 2.5% glutaraldehyde for 12 h at 4° C, rinsed in Phosphate‐Buffered Saline (PBS), and dehydrated in an ethanol gradient (30%–100%). After dehydration, disks were immersed in hexamethyldisilazane (HMDS), air‑dried, and sputter‑coated with gold‑palladium. Images were acquired to evaluate biofilm morphology.

### 2.9 Statistical Analysis

Statistical analyses were performed using SigmaPlot 12.0 (Systat Software Inc.). Data normality was assessed using the Shapiro–Wilk test, and homogeneity of variances was evaluated with Levene's test. Based on these assumptions, flexural strength data were analyzed using the Kruskal–Wallis test followed by Dunn's post hoc test, as normality criteria were not met. Microhardness data, which satisfied parametric assumptions, were analyzed using one‐way ANOVA followed by Tukey's post hoc test. The level of statistical significance was set at *α* = 0.05.

## 3 Results

Milled hybrid PMMA exhibited the lowest mean flexural strength (63.30 ± 8.27 MPa). However, this value did not differ significantly from either the 3D printed resin with inorganic filler (69.10 ± 5.87 MPa) or the 3D printed resin without inorganic filler (73.10 ± 2.51 MPa), indicating comparable performance among these three materials (Table 2). Although milled conventional PMMA demonstrated an intermediate flexural strength (79.70 ± 14.03 MPa) compared to the other groups, no statistically significant difference compared to 3D printed resins or milled hybrid PMMA was found. The composite resin (control group) presented the highest flexural strength (136.60 ± 26.15 MPa), significantly outperforming all other materials evaluated (*p* < 0.001).

**Table 2:** Means and standard deviations of flexural strength (MPa) and microhardness (HK) obtained for each experimental group.

Material/group	Flexural strength	Microhardness
3D printed resin with inorganic filler	69.10 (5.87)^B^	15.20 (1.94)^BC^
3D printed resin without inorganic filler	73.10 (2.51)^B^	12.66 (0.83)^C^
Composite resin	136.60 (26.15)^A^	32.75 (4.66)^A^
Milled conventional PMMA	79.70 (14.03)^AB^	16.03 (1.35)^BC^
Milled hybrid PMMA	63.30 (8.27)^B^	16.67 (2.10)^B^

*Note:* Different superscript letters within the same column indicate statistically significant differences among groups (Dunn's post hoc test, *α* = 0.05). Groups sharing at least one superscript letter are not significantly different (*p* > 0.05).

In regards of Vickers microhardness, the 3D printed resin without inorganic fillers showed the lowest hardness value (12.66 ± 0.83 HK). 3D printed resin with inorganic fillers (15.20 ± 1.94 HK) and milled conventional PMMA (16.03 ± 1.35 HK) exhibited higher values. However, none of them differed statistically from the 3D printed resin without inorganic fillers (*p* = 0.064). Conversely, milled hybrid PMMA (16.67 ± 2.10 HK) demonstrated significantly higher microhardness than the printed resin with inorganic fillers (*p* < 0.001). As expected, the composite resin showed the highest hardness among all groups (32.75 ± 4.66 HK), with statistically superior values compared with all other materials (*p* < 0.001).

**Figure 1:**
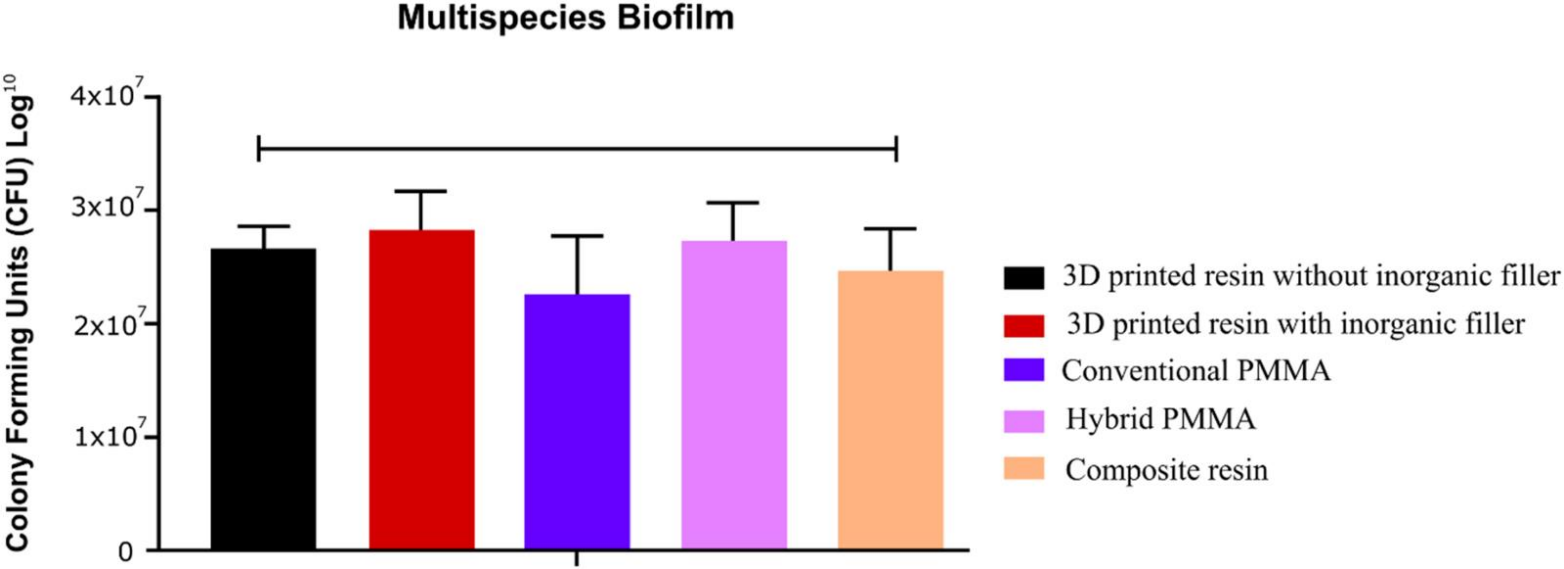
Multispecies biofilm growth on the temporary restorative materials. CFU/mL values (Log_10_ scale) after 7 days of biofilm formation. No statistically significant differences were observed among groups (*p* > 0.05).

**Figure 2:**
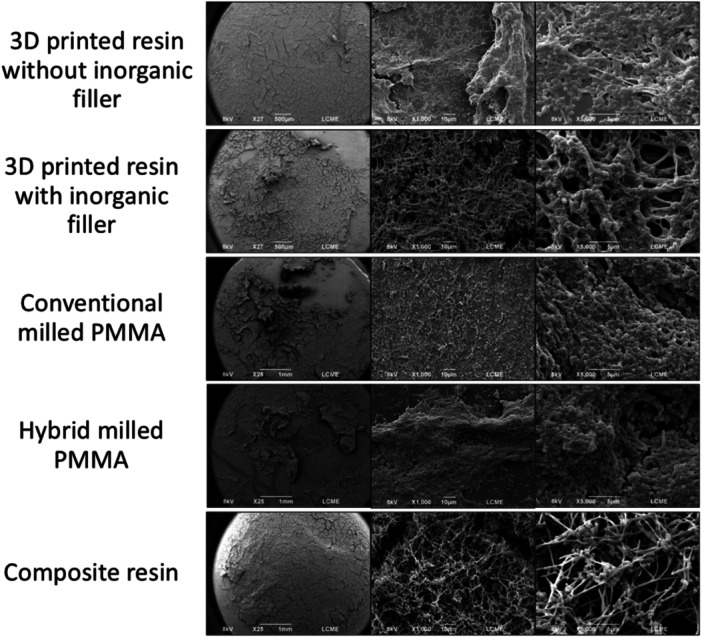
SEM micrographs of material surfaces after biofilm formation. Column 1: general surface overview; Column 2: 1000× magnification; Column 3: 5000× magnification. SEM, scanning electron microscopy.

Quantitative analysis of multispecies biofilm formation revealed no statistically significant differences among all temporary materials tested (*p* > 0.05). Average colony‑forming unit counts (CFU/mL), expressed on a logarithmic scale (Log_10_), ranged from 2.26 × 10^7^ to 2.82 × 10^7^ CFU/mL across groups (Table 3 and Figure 1). However, SEM imaging confirmed biofilm formation on all tested material surfaces, with noticeable variations in extracellular matrix density and morphological organization. Both tested3D printed resins (with and without inorganic fillers) exhibited dense, continuous biofilm coverage with abundant extracellular polymeric matrix. Milled hybrid PMMA specimens showed a more compact and organized biofilm structure, whereas the milled conventional PMMA displayed less dense bacterial aggregates with a less structured extracellular matrix. Composite resin exhibited pronounced bacterial colonization and a well‑defined fibrillar biofilm architecture, suggestive of increased adhesion and maturation (Figure 2).

**Table 3:** Means and standard deviations of colony‑forming unit quantification (CFU/mL) obtained for each experimental group.

Material/group	Average CFU/mL	SD
3D printed resin with inorganic filler	2.82 × 10^7^	2.1 × 10^6^
3D printed resin without inorganic filler	2.67 × 10^7^	1.0 × 10^6^
Composite resin	2.45 × 10^7^	3.1 × 10^6^
Milled conventional PMMA	2.75 × 10^7^	2.6 × 10^6^
Milled hybrid PMMA	2.26 × 10^7^	3.7 × 10^6^

Despite these qualitative morphological differences, no significant variation in CFU/mL was detected among the groups. This finding highlights the complementary value of SEM analysis, which can reveal structural differences in biofilm development that are not captured by quantitative microbiological assays alone.

## 4 Discussion

This study underscores the value of direct comparative assessments among CAD/CAM and 3D printed materials used for temporary restorations, with emphasis on their mechanical properties and biological behavior. Unlike most reports that benchmark CAD/CAM materials against acrylic or bis‑acrylic resins, we selected a nanofilled composite resin as a gold standard reference. This choice aligns with clinical practice, since composite resins are traditionally considered a high‑performance standard in restorative dentistry. To our knowledge, no prior study has evaluated, in a single design, the same combination of 3D printed resins (with and without inorganic fillers), milled PMMA (conventional and hybrid), and a composite resin, highlighting a relevant gap in the literature.

Among the CAD/CAM temporary materials, milled hybrid PMMA showed significantly higher Vickers microhardness (16.67 ± 2.10 HK) values than the 3D printed resin without inorganic filler (12.66 ± 0.83 HK), consistent with the general trend that milled materials often exhibit greater hardness than 3D printed counterparts (Jain et al. 2022). In contrast, regarding flexural strength, only milled conventional PMMA reached an intermediate‑to‑higher performance (79.70 ± 14.03 MPa), whereas milled hybrid PMMA yielded the lowest mean value (63.30 ± 8.27 MPa), even below both 3D printed resins. Possible reasons for such findings could related to differences in organic matrix composition, degree of cross‑linking, and filler content/distribution, all of which govern crack initiation, stress transfer, and energy dissipation (Liang et al. 2025).

With respect to microhardness, the absence of a statistically significant difference between milled conventional and hybrid PMMA was unexpected given the high silica content (30%–45%) reported for the hybrid. While filler addition typically elevates hardness, high nanosilica content can provoke agglomeration and thick interfacial layers, promoting structural heterogeneity, impairing stress transfer, and creating local weak zones, thereby limiting the expected hardness gains (Balos et al. 2020, 2014). Notably, specific mechanical data for this commercial hybrid PMMA are scarce, hindering direct cross‑study comparisons.

For the 3D printed resins, inorganic filler addition (~13%) yielded a numerically higher hardness (15.20 ± 1.94 HK) relative to the 3D printed resin without inorganic filler (12.66 ± 0.83 HK), suggesting a positive effect on surface hardness (Abad‐Coronel et al. 2025). However, this benefit did not extend to flexural strength, where the 3D printed without inorganic filler performed numerically better (73.10 ± 2.51 MPa) than with inorganic filler (69.10 ± 5.87 MPa). A similar pattern was noted for the milled groups: filler addition was not synonymous with improved flexural behavior. This divergence aligns with the concept that interfaces between filler and matrix may act as stress concentrators when coupling or dispersion is suboptimal, facilitating crack initiation and propagation under load (Debnath et al. 2004). Collectively, these results support an integrated evaluation of materials in which matrix chemistry, filler content/morphology, and interfacial engineering are considered together rather than in isolation. As expected, the composite resin outperformed all temporary materials across mechanical outcomes. Its behavior can be attributed to both the high filler loading and the engineered nano‑zirconia/silica system with effective matrix–filler integration (Hong et al. 2020; Yang et al. 2020). The resulting dense, homogeneous internal structure with reduced porosity and efficient stress distribution supports superior flexural strength and microhardness (Darbandi and Amin 2024). Consistent with that, performance in such systems reflects not only filler quantity but also uniform dispersion and optimized interfacial coupling (Hong et al. 2020; Yang et al. 2020).

No significant differences were detected in multispecies biofilm CFU among the temporary materials, suggesting that, under the experimental conditions, composition and manufacturing route exerted limited influence on overall microbial load. A likely contributor to this convergence was the application of glaze after polishing to both milled and 3D printed specimens, which may have sealed and standardized the surface topography, leading to similar adhesion conditions. Although milled materials are often more compact and less porous than 3D printed ones, (Jain et al. 2022; Mahran et al. 2025) this did not translate into lower CFU here, reinforcing that for mature biofilms extrinsic factors (salivary flow, nutrient availability, exposure time) can be more decisive (Souza et al. 2024). The interpretation of biofilm results must be approached with caution due to the presence of a confounding variable, as glaze was not applied to the composite resin group. Surface coating is known to significantly influence bacterial adhesion, and therefore direct comparisons between groups may not fully reflect intrinsic material properties. In the 3D printed resins (with and without inorganic filler), a thicker extracellular matrix was observed, potentially indicating greater maturation, possibly influenced by higher roughness or less hydrophobic matrix chemistry, as reported for certain 3D printed denture base resins (Carneiro Pereira et al. 2024). Clinically, a dense extracellular polymeric matrix affords increased protection against antimicrobials (Flemming et al. 2016) and complicates mechanical biofilm removal (Stoodley et al. 2002).

This study presents some limitations that should be considered when interpreting the findings. First, the lack of uniform surface treatment across all groups, particularly the absence of glaze in the composite resin group, introduces a potential confounding factor in the biological analysis. However, this approach was intentionally adopted to better reflect clinical practice, as composite resins are typically finished and polished chairside without the application of a glaze layer, unlike many CAD/CAM and 3D‐printed provisional materials for which surface coatings are manufacturer‐recommended. Consequently, the microhardness values reported for the glazed materials may reflect not only the intrinsic properties of the base material but also the contribution of the glaze layer. Likewise, the glaze may have influenced surface characteristics relevant to biofilm adhesion. Although this limits direct comparison of the underlying materials, it improves the clinical relevance of the findings by reproducing the surface condition under which these materials are commonly used in practice. Second, the microbiological model was simplified, using a single donor and controlled laboratory conditions, which may not fully reproduce the complexity of oral biofilms (Lemus and Valm 2023). Future studies should incorporate selective media, controlled oxygen atmospheres, and molecular identification (e.g., PCR, mass spectrometry) to expand ecological resolution. Third, the relatively small sample size for CFU quantification may limit statistical power. Finally, the inherent variability of 3D‑printing parameters (build orientation, post‑cure protocol, device technology) was not explored here; our focus was comparative material composition.

The null hypothesis stated that temporary materials manufactured by 3D printing or milling would not perform worse than composite across all evaluated variables. The null hypothesis has rejected considering the assessed mechanical properties (flexural strength and microhardness), but was accepted in regards of biofilm quantification, where no significant differences emerged. Thus, the hypothesis was partially supported.

## 5 Conclusions

Within the limitations of the present study, it can be concluded that:

– 3D printed and milled PMMA temporary materials exhibited comparable flexural strength and microhardness to one another, whereas the composite resin demonstrated superior values for both properties.
– No differences were detected under the present experimental conditions for the quantitative CFU among temporary materials under the present conditions; however, SEM revealed morphological differences in extracellular matrix architecture across groups.

## Author Contributions

**Indyara Cerutti:** Conception and design of the study, acquisition of data, or analysis and interpretation of data, drafting the article or revising it critically for important intellectual content, final approval of the version to be submitted. **Juliana S. R. de Andrade:** Acquisition of data, interpretation of data, drafting the article or revising it critically for important intellectual content, final approval of the version to be submitted. **Andressa da Silva Barboz:** Acquisition of data, interpretation of data, drafting the article or revising it critically for important intellectual content, final approval of the version to be submitted. **Jaqueline B. Machado:** Acquisition of data, interpretation of data, drafting the article or revising it critically for important intellectual content, final approval of the version to be submitted. **Dewey Du‐Hyeong Lee:** Interpretation of data, drafting the article or revising it critically for important intellectual content, final approval of the version to be submitted. **Mateus Bertolini Fernandes dos Santos:** Conception and design of the study, analysis and interpretation of data, drafting the article or revising it critically for important intellectual content, final approval of the version to be submitted.

## Conflicts of Interest

The authors declare no conflicts of interest.

## Data Availability

Data available on reasonable request from the authors.
